# Application of Bayesian methods to accelerate rare disease drug development: scopes and hurdles

**DOI:** 10.1186/s13023-022-02342-5

**Published:** 2022-05-07

**Authors:** Kelley M. Kidwell, Satrajit Roychoudhury, Barbara Wendelberger, John Scott, Tara Moroz, Shaoming Yin, Madhurima Majumder, John Zhong, Raymond A. Huml, Veronica Miller

**Affiliations:** 1 Department of Biostatistics, University of Michigan School of Public Health, Ann Arbor, MI USA; 2 Pfizer Inc., New York, NY USA; 3 Berry Consultants, LLC, Austin, TX USA; 4 Food and Drug Administration, Washington, DC USA; 5 Takeda Pharmaceutical Company Limited, Cambridge, MA USA; 6 Daiichi Sankyo Inc., Basking Ridge, NJ USA; 7 REGENXBIO Inc., Rockville, MD USA; 8 Syneos Health Clinical Solutions, Morrisville, NC USA; 9 Forum for Collaborative Research, University of California School of Public Health, Berkeley, CA USA

**Keywords:** Small sample, Clinical trial, Prior distribution, External control, Meta-analytic predictive approach, SMART, Platform, Adaptive

## Abstract

**Background:**

Design and analysis of clinical trials for rare and ultra-rare disease pose unique challenges to the practitioners. Meeting conventional power requirements is infeasible for diseases where sample sizes are inherently very small. Moreover, rare disease populations are generally heterogeneous and widely dispersed, which complicates study enrollment and design. Leveraging all available information in rare and ultra-rare disease trials can improve both drug development and informed decision-making processes.

**Main text:**

Bayesian statistics provides a formal framework for combining all relevant information at all stages of the clinical trial, including trial design, execution, and analysis. This manuscript provides an overview of different Bayesian methods applicable to clinical trials in rare disease. We present real or hypothetical case studies that address the key needs of rare disease drug development highlighting several specific Bayesian examples of clinical trials. Advantages and hurdles of these approaches are discussed in detail. In addition, we emphasize the practical and regulatory aspects in the context of real-life applications.

**Conclusion:**

The use of innovative trial designs such as master protocols and complex adaptive designs in conjunction with a Bayesian approach may help to reduce sample size, select the correct treatment and population, and accurately and reliably assess the treatment effect in the rare disease setting.

**Supplementary Information:**

The online version contains supplementary material available at 10.1186/s13023-022-02342-5.

## Background

The Orphan Drug Act defines a rare disease as one that affects less than 200,000 individuals in the United States, while the European Union defines a rare disease as one that affects 1 per 2000 individuals or fewer [1]; combined there are somewhere between six to ten thousand rare diseases [2]. Although individually rare, collectively, rare diseases affect 400–700 million persons globally at any point in time [3]. However, fewer than 10% of rare diseases have approved treatments leaving an urgent unmet medical need for rare disease patients and their families [4]. A 2018 review from the US, EU, and Japan reports 28,526 clinical trials studying 1539 drugs for 1535 rare diseases; over half (51%) of these trials were in the setting of rare cancers [5]. Although clinical trials are underway, there are unique challenges to rare disease research such that trials investigating drugs for rare diseases tend to enroll fewer patients, are more likely to be non-randomized, and are more often open label than non-rare disease trials [6].

A main challenge in drug development for rare diseases is linked to the challenge of generating robust evidence from clinical trials with limited sample size. Certain diseases are so rare that enrolling a reasonable number of patients is difficult when patients are recruited worldwide and over several years. Results from any such small trial enable limited interpretation of the treatment effect due to the lack of precision. Small participant numbers tend to correspond to a small number of endpoint events. This situation is often referred to as the “zero-numerator problem” [7]. Traditional statistical methods yield overly conservative results in such situations. Given these challenges, alternative design choices that ensure utilization of all available (trial internal and external) data on treatment efficacy and safety should be considered to maximize the ability to draw clinically relevant conclusions. Bayesian methods have been suggested as a framework to investigate interventions in small samples. Bayesian methods provide an intuitive probability that the treatment effect lies in an effective range which has important clinical interpretability and can provide more practical results when studying treatments in small samples [8–11]. These methods have gained significant interest in clinical trials generally [12–17], particularly rare disease trials [8, 17–21]. Yet, Bayesian methods have been criticized for the subjective aspects of selection of prior distributions that summarize our previous knowledge, lack of software for efficient implementation, and the necessary time, effort and incentive for statisticians and clinicians required to learn, implement and interpret Bayesian methods [22].

Small population clinical trials have been the focus of much methodological research activity, regulatory guidelines, and patient advocacy in the last two decades [23]. However, real-life applications of novel methods are still rare [24]. In this paper, we present the usefulness of Bayesian design and analyses in rare disease settings while acknowledging the complexity and potential issues of such a framework. This paper is unique as it presents real or hypothetical case studies that address the key needs of rare disease drug development highlighting several specific Bayesian examples of clinical trials. These examples illustrate the elicitation of prior distributions using external data, development of the Bayesian model to address key questions of clinical interest, process of simulations to describe frequentist operating characteristics, and the presentation and communication of results. Additionally, we include code to implement some of the methods as the Additional file 1. The goal is not to derive the statistical intricacies of Bayesian trials, but to show utility of the approaches with concrete examples. This manuscript will help a broad set of researchers to feel empowered while implementing these designs and analyses and improve treatment selection for patients with a rare disease.

The paper is structured as follows. In “Utility of Bayesian methods to address design and analysis challenges in rare disease” section, we highlight the aspects of Bayesian statistics helpful for rare disease clinical trials. A number of examples are discussed in “Case studies illustrating applicability and execution of Bayesian methods” section to show its utility in real-life. “Implementation aspects of using Bayesian methods in rare disease trials” section discusses some operational aspects followed by a concluding discussion.

## Main text

### Utility of Bayesian methods to address design and analysis challenges in rare disease

Clinical trials have historically been analyzed using frequentist statistics. Frequentist analysis depends heavily on a frequency definition of probability based on long-run properties of repeated experiments, which is grounded in the concept of the p-value. Challenges of frequentist analysis include difficulty comprehending the proper meaning of the p-value and confidence intervals, inability to estimate the probability of clinical benefit, and no straightforward mechanism for combining external information with internal trial data [25, 26]. Bayesian statistics are inherently well-suited to address these challenges.

Bayesian statistics are particularly relevant in rare disease research because many trials are too small to show nominal statistical significance. As opposed to the focus on p-values, Bayesian statistics provide the probability of a clinically meaningful treatment benefit (e.g., the probability that treatment A has at least a 10% greater response rate than treatment B is 85%). This probability directly addresses the key scientific question regarding the benefit of treatment A over treatment B and is appealing to clinicians, patients, and other stakeholders.

**Fig. 1:**
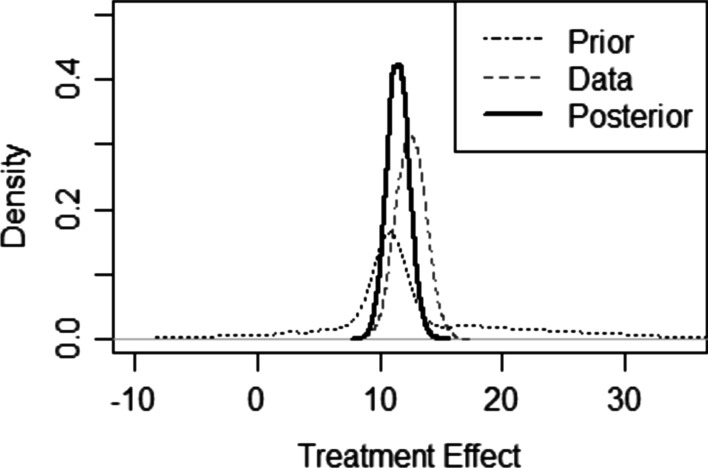
Bayesian statistics provide a systematic approach to combine all available evidence. The prior illustrates knowledge known before the trial and is based on historical data from old trials, published literature, ongoing trials, and other real-world data, while the data is collected during the current clinical trial and provides the likelihood of the treatment effect. The prior and data are combined to produce the updated information or posterior distribution of the treatment effect, which is used to quantify results and infer conclusions. For example, here we see from the posterior distribution that there is 99% probability that the treatment effect is greater than 10

Moreover, the Bayesian approach provides a formal framework to incorporate external information into the statistical analysis of a clinical trial. External information includes, but is not limited to, historical data from previous trials, published literature, ongoing trials, and other real-world data [27]. There is an intrinsic interest in, and need for, leveraging all available information in rare disease clinical trials to ensure an efficient design and analysis. Including external information is ethically appealing as it allows for trials with smaller sample sizes or unequal randomization (e.g., more subjects on treatment than control) and enables a single, otherwise underpowered trial to provide sufficient information for robust decision-making. Efficient designs also help prevent the erroneous abandonment of an intervention for which confident conclusions about efficacy require additional data. Applications of using external data in clinical trials are seen in earlier phases of drug development [28–30], occasionally in Phase III trials [31], and in special areas such as medical devices (FDA, 2010a, [32]), orphan indications [33] and extrapolation in pediatric studies [34]. Recently, the 21st Century Cures Act [35] and Prescription Drug User Fee Act (PDUFA) VI [36] encouraged the use of complex clinical trial design, including Bayesian designs. Several recent regulatory documents [37–39] discussed the opportunities and challenges of using external information in trial design and analysis. External data and/or expert opinion are first used to construct a *prior* distribution of the parameter of interest (see Fig. 1). Then, the current trial data (summarized with a mathematical function known as the *likelihood*) is analyzed using the *prior,* and a corresponding *posterior distribution* (i.e. updated information) is then used for trial reporting and decision making. The Bayesian approach aligns with clinical practice in the sense that results from a Bayesian trial are interpreted considering prior knowledge based on known mechanisms of effect and previously available information. In the next subsection we discuss specific examples of Bayesian methods for the design and analysis of rare disease clinical trials to further illustrate their utility.

### Case studies illustrating applicability and execution of Bayesian methods

Here, we illustrate the usefulness of a Bayesian approach through specific examples in rare disease trials. We focus on the use of external control data through the meta-analytic prior, multi-stage designs, platform studies, and disease progression modelling. Other areas where Bayesian methods are applicable to rare disease research include seamless Phase II-III studies [40, 41] and Phase II proof of concept studies [42, 43]. The studies we present here are either hypothetical, based on ideas ongoing at the time this manuscript was written, or have ended and published results.

#### Use of external control data in the designing phase III trial for Progressive Supranuclear Palsy (PSP)

We present a hypothetical pivotal Phase III design of a new drug in Progressive Supranuclear Palsy (PSP). The goal of this hypothetical trial is to reduce the number of subjects in the placebo arm using data from three completed randomized studies and increase efficiency. The design uses a meta-analysis-based approach with additional considerations such as practical feasibility and regulatory requirements. PSP is a degenerative neurological disorder that causes progressive impairment of balance and walking; impaired eye movement, especially in the downward direction; abnormal muscle tone; speech difficulties; and problems related to swallowing and eating. The PSP Rating Scale (PSPRS) is a disease specific quantitative measure of disability and attempts to include all the important areas of clinical impairment in PSP [44–46]. It measures disability across 28 items in six domains: daily activities (by history), behavior, bulbar, ocular motor, limb motor and gait/midline. Mean change from baseline PSPRS score at week 52 is accepted as a primary outcome measure of a clinical trial by clinicians and regulatory agencies when evaluating a new therapy for PSP. A four-point improvement over placebo in mean change from baseline at 52 weeks (treatment effect δ = 4) is considered clinically meaningful. A traditional frequentist design with 1:1 randomization to a placebo and treatment arm would require 85 patients per arm to conduct an adequately well controlled trial (one-sided α = 2.5%, power = 90%, mean change from placebo = 4, assumed standard deviation = 8). We explore an alternative Bayesian design with 2:1 randomization in favor of the experimental treatment (85 for experimental therapy arm and 43 for the placebo arm) which is ethically and practically appealing as this helps to reduce the number of subjects in the placebo arm.

An informative prior for the mean change in PSPRS at week 52 for the placebo arm is derived using placebo data from the three randomized Phase II studies conducted previously in PSP and the meta-analytic-predictive (MAP) approach [47, 48]. The MAP framework provides a mathematical structure to link parameters of interest in the external data (e.g., mean change in PSPRS at week 52 for the placebo arm in three phase II trials) and use the extrapolation principle to predict the possible outcomes for the current trial which can be used as a prior in the analysis. The key assumption of this approach is *exchangeability* or *similarity* of external control information with the current trial. Exchangeability enables the current trial to ‘borrow strength’ from external data sources using a *hierarchical model* which takes into account the heterogeneity between current trial and external data. The overall degree of borrowing depends on the variance parameter of the hierarchical model, which is known as the “between-trial data source heterogeneity parameter.” Specification of this variance parameter requires special attention in practice to ensure an appropriate result. This is especially important when the number of external trials is small. We suggest a weakly informative prior for between-trial heterogeneity that covers a wide range of plausible endpoint outcomes and the variability associated with it [12, 49, 50].

The three Phase II studies used as external information in this example are similar in terms of population, inclusion/exclusion criteria, and other important study characteristics. Table 1 summarizes the mean change in PSPRS score for the placebo group in the three trials. Using the MAP approach, the predictive distribution for the mean change in PSPRS score at week 52 in a new study is derived, leading to an estimated mean of 10.8 (and a 95% credible interval of 8.4 to 13.4). For ease of use and interpretation, we approximate this predictive distribution assuming a normal density with matching mean and standard deviation for this example. The result of this process is the MAP prior with a normal (mean = 10.8, sd = 1.19) distribution. Alternatively, a mixture of normal distributions can be used to approximate the predictive distribution [39] accurately. Further details of the MAP prior derivation are included in the Additional file 1.

**Table 1:** Available Phase II data for the control group for a hypothetical pivotal Phase III design of a new drug in Progressive Supranuclear Palsy

Study	N	Mean change from baseline to week 52 in PSPRS	Standard error
Boxer et al. [51]	153	10.9	0.99
Tolosa et al. [52]	31	11.4	1.13
Höglinger et al. [53]	59	10.5	1.00

PSPRS is the PSP Rating Scale (PSPRS) which is a disease specific quantitative measure of disability

Although the external and new populations are assumed to be similar or exchangeable to allow one of them to inform the other, they are not the same and important differences, not known a priori, might exist. Therefore, along with informative priors, we also need to incorporate a certain degree of skepticism. This can be achieved using robust mixture priors which is a combination of informative and non-informative priors [48]. It allows for *dynamic borrowing* of prior information; the analysis learns how much of the external data to borrow in the prior based on the consistency between the external information and the trial data. Mixture priors have been proposed in different contexts (e.g., bridging studies, historical controls, pediatric extrapolation) and are relevant for rare disease studies. Further robustification of the MAP prior in our PSP example to handle conflict between external and trial placebo data is provided by including a non-informative Normal (mean = 15, sd = 10) prior with 50% weight to the MAP prior to reflect considerable heterogeneity between the Phase II and Phase III population and to control inflation of the false positive rate. This prior is used as the historical data prior for the placebo group in the primary analysis. As no relevant external information is available for the experimental treatment, a non-informative prior (normal distribution with parameters mean = 0 and sd = 10) is used for mean change from baseline to week 52 in PSPRS for the experimental treatment group.

**Fig. 2:**
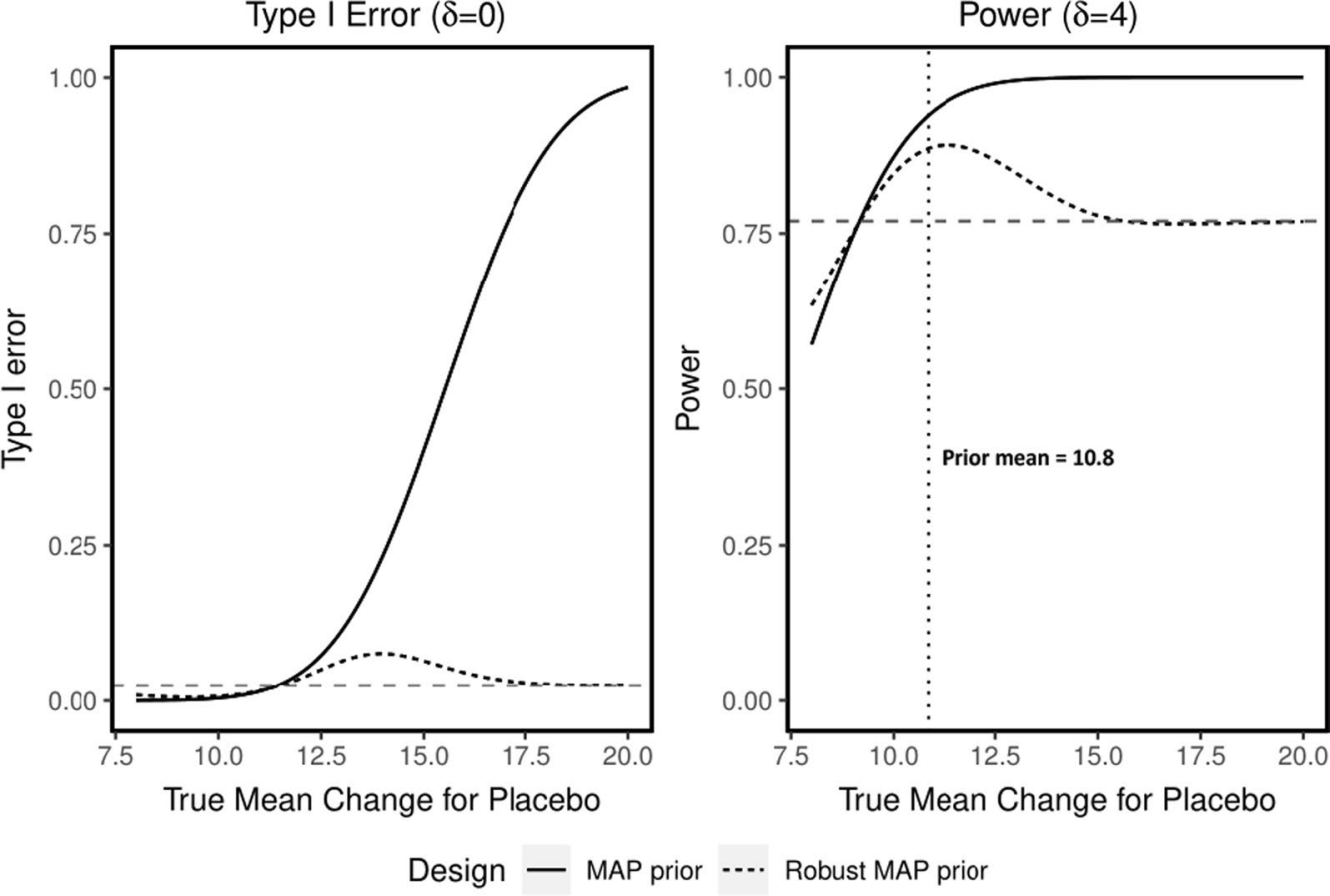
Frequentist Operating Characteristics (type I error (left panel) and power (right panel) of proposed design with meta-analytic predictive (MAP; solid line) and robust MAP (dotted line) priors under different scenarios for mean change from baseline in PSPRS at week 52 between treatment and placebo arms (δ). When δ = 0 (left panel) there is no difference between placebo and treatment, whereas when δ = 4 (right panel) there is a treatment difference. The plot also shows the type I error (0.025, left panel) and power (0.8, right panel) for the traditional frequentist design (grey dashed line)

In a clinical trial setting, an important step of design is assessing the operating characteristics; typically, this has included frequentist concepts such as type I error and power [15]. All analyses for our example were conducted in R version 4.0 [54] with the package RBesT 1.6.1 [55] (see Additional file 1: Appendix 1 for the detailed implementation); computations are very fast using RBesT due to using an analytical method rather than time consuming simulations. Figure 2 represents type I error and power of the proposed design with and without a robust MAP prior. Study success is declared if there is considerable chance of a positive treatment effect (P(δ > 0|data) > 0.975), where δ represents the difference in mean change from baseline in PSPRS at 52 weeks between treatment and placebo group.

In the left panel of Fig. 2, we see that, in contrast to the (informative) MAP prior (solid line), the robust MAP prior (dotted line) does not lead to an excessive increase of type I error when there are conflicts between trial placebo data and external sources. The maximum type I error is 6.3%. For power (right panel of Fig. 2), the MAP prior and the robust MAP prior offer considerable gains compared to the traditional frequentist design (grey horizontal dashed line) with the same sample size on the treatment arm, if the true mean change in PSPRS is in the range of prior support (true mean change PSPRS at 52 weeks between 9 and 13). The power gain is impressive (as high as 14%) compared to the traditional design if external placebo and trial placebo data are consistent. In case of conflict (true mean change PSPRS at 52 weeks > 12), the inflation of type I error for the MAP prior is clearly much stronger compared to the robust version. For very clear prior-data conflict (true mean change PSPRS at 52 weeks > 14), the power of the robust MAP prior is approximately the same as the traditional design.

In summary, this example suggests that a reduced sample size design with robust MAP priors containing a weakly informative component has good robustness properties and has improved power in some scenarios when compared to a more traditional frequentist design. The definition of acceptable frequentist metrics needs to be reconsidered for each unique trial setting. This includes careful judgment about how likely scenarios of conflict are. Other approaches include the *power prior* [56] and *commensurate prior* [57]. MAP prior, commensurate prior, and power prior share the same general feature that they discount historical data to account for heterogeneity between external and trial internal information.

#### Multi-stage Bayesian studies

**Fig. 3:**
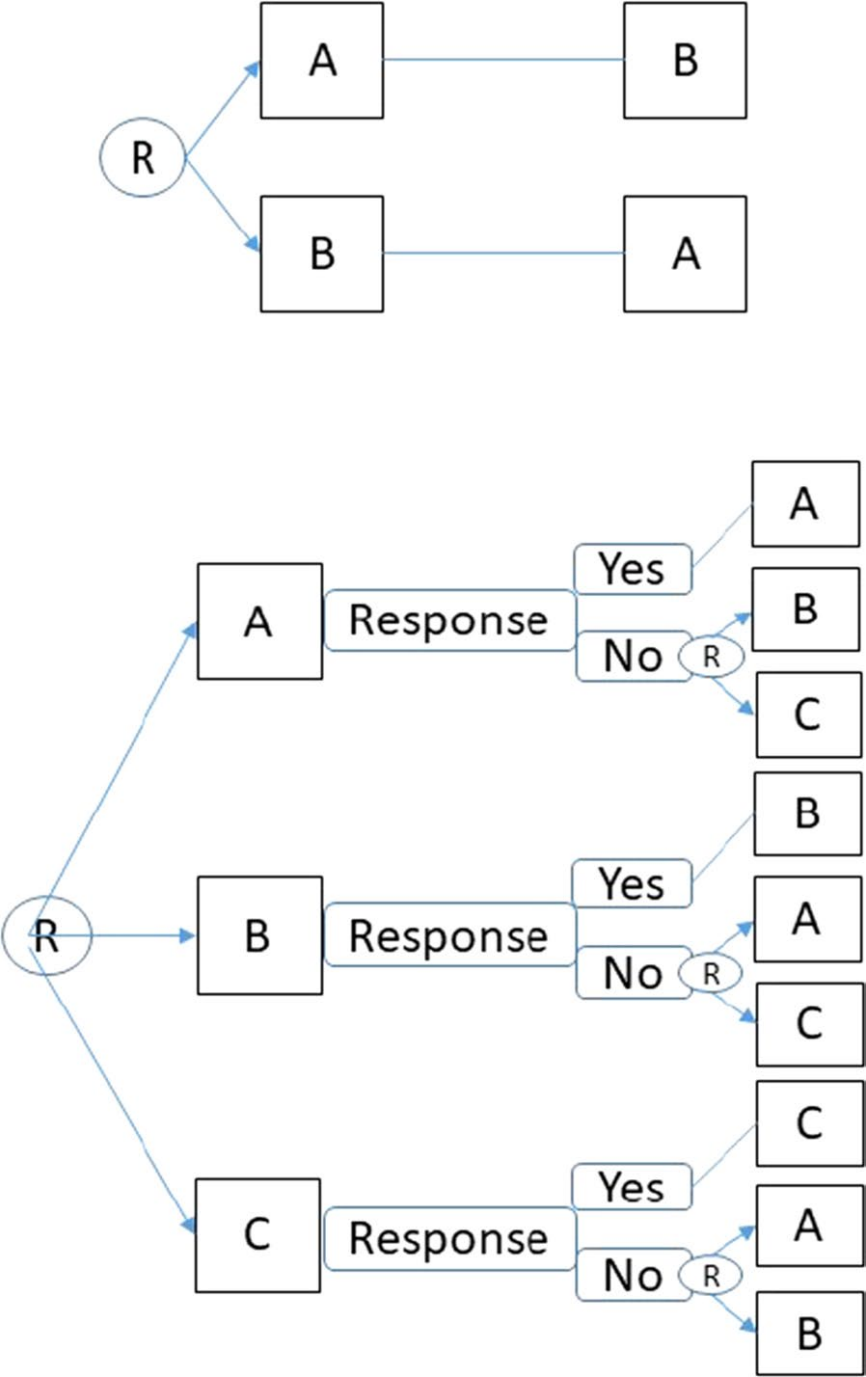
Top panel: shows a 2 × 2 crossover design where a group of participants are randomized to a sequence of treatments to first receive treatment A then B or first receive B then A. Bottom panel: shows a small n, sequential, multiple assignment, randomized trial design with three treatment options like the ARAMIS design. R denotes randomization and A, B, C denote intervention options

Emerging research has applied sequential, multiple assignment, randomized trial (SMART) designs in rare diseases using the Bayesian framework for analysis to obtain efficient treatment estimates. Two ongoing, international, Phase III trials, the International Penile Advanced Cancer Trial (InPACT, NCT02305654) [58] and A Randomized Multicenter study for isolated skin vasculitis (ARAMIS, NCT02939573) [59, 60] are examples of small sample SMART designs. We note these small sample SMART designs are motivated by different goals and analyzed differently than standard SMART designs [61, 62]. A small sample (n) SMART (snSMART) design (see Fig. 3, bottom) is similar to a crossover design (see Fig. 3, top), but treatment assignment or re-randomization in the second stage is restricted based on high risk or non-response to the initial treatment received in the first stage. Restricting crossover to only those who do not respond to stage 1 treatment may further increase recruitment and reduce issues with non-adherence or dropout over that of standard crossover trials. Multiple periods or stages of treatment within a trial can provide within-person treatment differences, address multiple questions of interest and/or provide more data from a small sample. Both InPACT and ARAMIS employ Bayesian approaches and plan to report the posterior distribution to infer about treatment effects, no hypothesis testing is planned.

InPACT aims to enroll 400 men in their two-stage design with the first stage randomization to one of three arms: no neoadjuvant therapy, neoadjuvant chemotherapy, and neoadjuvant chemoradiotherapy. After lymph node dissection, if patients are considered high risk for recurrence, they enter the second stage randomization to chemoradiotherapy alone or prophylactic pelvic lymph node dissection plus chemoradiotherapy. InPACT applies re-randomization in the design to address two main questions of interest: (1) if neoadjuvant therapy before surgery improves survival and if so, if chemotherapy or chemoradiotherapy is the best option and (2) for those who have a high risk of recurrence after surgery, if prophylactic pelvic lymph node dissection plus chemoradiation to the groin and pelvis improves survival over chemoradiation alone. The statistical analysis plan of InPACT proposes primary analyses estimating the posterior distribution for the hazard ratios using non-informative priors with secondary analyses using evidence-based priors such as those developed in the PSP example using previous trial data.

ARAMIS is a smaller two-stage trial that aims to enroll 90 individuals with the first randomization to one of three active treatment arms. After 6 months, the patients are assessed for response to treatment and the non-responders are re-randomized to one of the two treatments that they did not initially receive and are followed for an additional 6 months. ARAMIS applies re-randomization to obtain more information from the small number of individuals in the trial so that outcomes (response rate) can be combined across the two stages of the trial for more efficient first stage treatment effect estimates. Originally, ARAMIS was planned assuming a frequentist analysis ignoring the second stage data from some participants [60], however, the protocol was modified given development of more efficient Bayesian methods that pool information across two stages to estimate the first stage treatment effects for the three treatments of interest [63]. The revised analytic approach for ARAMIS places evidence- and expert-opinion-based prior distributions across model parameters including the treatment response rates and parameters in the model that link the stage 1 and stage 2 outcomes to estimate the posterior distributions of the first stage treatment effects. Additional methods for snSMART design variations have been developed that apply the Bayesian approach to a two-stage design to more efficiently estimate the treatment effect of interest [64–68].

#### Bayesian platform studies

Other types of multi-arm designs can be described by master protocols including umbrella or platform trials that focus on one disease but investigate several different experimental drugs targeting different biomarkers or genetic aberrations and basket trials [69–71] that typically test one targeted therapy across multiple diseases. Master protocols have mostly been used in the oncology setting but could be especially useful for a wide variety of rare diseases [72]. Umbrella or platform studies may have one shared control arm and multiple experimental arms or include multiple standard of care arms. Platform trials incorporate more adaptations, for example modifying randomization probabilities to treatment(s) based on accruing information, than a standard umbrella design [73–75], and allow therapies to enter or leave the platform based on emerging treatments. These multi-arm designs have been recommended for rare diseases based on a potential gain in efficiency through sharing the same control group, reducing the chance of assignment to placebo, comparing several treatments, the ability to pool data across treatments, and sharing trial resources [21]. Bayesian methods are able to provide these required flexibilities in the platform design [11].

Recently, a Bayesian platform trial, INHIBIT (NCT04303559), was proposed to investigate treatments to prevent and eliminate inhibitor formation in 156 individuals with hemophilia over 6 years [76]. INHIBIT is a platform trial that includes two Phase III studies, a non-inferiority design that studies the prevention of inhibitors (n = 66) and a superiority design that studies the eradication of inhibitors in patients with severe hemophilia A (n = 90). Two Phase III trials within one protocol capitalize on the same centers, labs and visit timing, while allowing other drugs to enter the trials if they emerge over the six years expected for recruitment.

Statistical efficiency is achieved using an informative prior distribution that incorporates relevant historical data, use of posterior distribution, time-to-event modeling to estimate the proportion of individuals who develop inhibitors or eliminate inhibitors within 48 weeks, and detailed simulation studies to identify operating characteristics. Information from a similar previous study is incorporated through an informative prior distribution for the standard of care treatment arm in the prevention study; this results in fewer individuals for that arm and smaller sample size for the trial (as similarly illustrated in the PSP example). Additionally, each trial includes one interim analysis after 75% of individuals have been enrolled for early decision making. This allows the trials to stop with even fewer individuals if there is a strong signal based on the high posterior probability of achieving superiority. The prevention trial is a non-inferiority trial (margin of 10%) that has been sized using a one-sided type I error rate of 5% and the eradication trial is a superiority trial (15% superiority) with a two-sided 5% type I error. The research team has illustrated the advantage in power of the Bayesian designs over frequentist designs, particularly in scenarios where there is a true treatment effect [76].

While the INHIBIT study design is intended to investigate inhibitor development among previously untreated patients, inhibitors also occur – much more rarely – among previously treated patients. In this setting, investigators could run into the z*ero-numerator* problem or the situation of estimating the probability of an event that is conceivably possible but has not yet occurred in the data that are available [77, 78]. Due to small sample size, rare disease trials often face the issue of zero incidents for some of the important efficacy and life-threatening safety outcomes which play an important role in the risk–benefit assessment of a new drug. Bayesian methods provide natural solutions to the problem of the zero-numerator problem. Though observing no occurrences of an event indicates low probability, it does not imply a zero chance. Therefore, there is scientific and regulatory interest in evaluating the upper limit of the chance of the occurrence of these important outcomes. One solution assumes a beta-binomial model [7, 77, 78] to facilitate the modeling of event data. The upper bound of the event rate is calculated using the 95th percentile of the posterior distribution of the rate under different informative prior distributions. The choice of the informative prior depends on several factors including available trial external data for standard of care and expert opinion [79]. The Bayesian approach enables the possibility of incorporating all available information in the prior while making inference about the rates of events of interest even with a small sample size.

#### Bayesian adaptive platform trial with disease progression modeling

Another multi-arm platform trial that incorporates innovative design and Bayesian methods is the Phase III Dominantly Inherited Alzheimer Network Trials Unit (DIAN-TU) adaptive platform study (NCT01760005) which opened in 2014. This platform study included two active treatment arms and a pooled, shared placebo arm to study treatments to slow or prevent cognitive decline from autosomal dominant Alzheimer’s disease (affects < 1% of Alzheimer’s population) [80]. Originally, enrollment was estimated at 210 participants, but was updated in October 2020 to 490. Mutation positive individuals were assigned to one of the two study arms and then randomized within that arm 3:1 to active drug. Mutation negative individuals were assigned to placebo and were not included in the primary analyses. This study combines a platform study design to study the effect of two drugs versus placebo, a shared placebo group, the use of natural history data to model disease progression, and is adaptive by including interim analyses to assess early efficacy within a Bayesian framework.

Natural history studies are important for understanding rare diseases—the cause(s), range of manifestations, and progression—as well as for raising awareness. Understanding disease progression as measured by clinical outcome measures over time is critical for successful clinical trial development. Clinical trial simulators based on disease progression models have been utilized to make evidence‐based decisions in clinical trial design. Recent guidelines [81, 82] from the US Food and Drug Administration (FDA) have emphasized the utility of natural history studies in rare disease drug development. In the setting of rare diseases, Bayesian models can be used to predict disease progression based on endpoints envisaged in the trial under consideration. In addition, the Bayesian framework provides a unified approach for inference (i.e., efficacy of the treatment in trial) and prediction (i.e., future endpoints for that patient or disease trajectory or treatment effect for a similar, given patient).

Using data from the DIAN observational study, the DIAN-TU team constructed flexible, disease progression models with random effects for the estimated age of onset of Alzheimer’s and the individual’s healthy cognitive score [83]. A hierarchical Bayesian disease progression model uses available data collected over time from a natural history study and permits the estimation of trajectories (i.e., the time effect) for each patient, as well as the evaluation of patient-to-patient variability and heterogeneity in these trajectories. The main idea of these models is to show the course of disease over time along with identifying important patient-related factors causing different progression patterns. Moreover, the use of a predictive distribution allows individualized prediction to identify possible treatment responders. The framework is flexible enough to incorporate different kinds of time effects including nonlinear trends. Like other predictive modeling, disease progression also requires appropriate planning to “train” the proposed model (i.e., examine the features of the model via simulations) and apply it during “test” time (i.e., within a clinical trial) [43, 84, 85].

Interim analyses were included in the trial design such that data were analyzed when the last participant reached two years and three years follow-up. At the interim analyses, the trial could stop for efficacy if the posterior probability that the treatment slowed the rate of cognitive decline was greater than or equal to 0.9952. Sequential designs, like the INHIBIT and DIAN-TU study, use accumulating data to make decisions at interim time points in a trial and are most useful when patient outcomes can be measured quickly relative to patient accrual which is typical of many rare disease studies. A trial can be stopped early based on Bayesian analyses that provide the probability of treatment efficacy. Alternatively, a trial can be stopped early for futility if there is a high probability that treatment will not be found to be effective. Pre-trial simulations must be performed to assess trial characteristics under a variety of real-life settings to decide if incorporating adaptive components is advantageous. Additional examples of Bayesian interim analyses in rare disease are the Focal Cerebral Arteriopathy Steroid (FOCAS) trial [86] and the study of Suvodirsen in ambulatory patients with Duchenne Muscular Dystrophy (DYSTANCE 51) [87]. There are also methods available for a Bayesian response adaptive design that allocates more participants to the best performing arm, has higher power than a traditional fixed design, and has small bias and mean squared error of the treatment effect estimates [88].

The DIAN-TU trial did not stop at an interim analysis and initial results on 194 participants with follow-up up to 7 years were shared at conferences in 2020 [89–91]. Neither drug resulted in slowing cognitive decline, although one drug was associated with changes in biomarkers related to the mechanism of cognitive change [92]. Although the drugs were not found to be efficacious, the platform study illustrates many advances in trial design and savings in power due to disease progression modeling and a shared placebo group.

### Implementation aspects of using Bayesian methods in rare disease trials

Despite the strong methodological groundwork and advantages in rare disease drug development, there are certain limitations in the uses of the Bayesian approach. As seen in the PSP example (Fig. 2), there is a potential increase in type I error when external control data and trial data have major conflict. This requires further calibration of the prior and/or a decision rule in confirmatory trials with more stringent error control requirements. In addition, the use of Bayesian trial design and analysis requires pro-active planning and discussion with regulators well in advance of the start of a trial. Finally, the Bayesian approach may pose an additional challenge regarding the communication of trial results to non-statisticians who are more familiar with traditional analysis with p-values. We elaborate on the following topics which require careful attention while using Bayesian methods in confirmatory trials: subjective choice information for the prior, computational burden to evaluate frequentist operating characteristics, lack of familiarity by non-statisticians, and longer time to review and level of scrutiny from regulatory authorities. We discuss some of these aspects and the efforts to overcome them.

#### Choice of prior distribution

The choice of a prior distribution is a well-known controversial topic in Bayesian analysis. Different choices of prior could lead to different conclusions about the trial, particularly in small sample settings such as rare diseases. In such situations the prior distributions may have larger impacts on the posterior distribution and trial conclusions. One option to derive the prior is to elicit expert opinion. This procedure may be useful to translate the available expert knowledge about the treatment effects into the prior probability distribution [93–96]. A systematic elicitation approach can limit some of the individual biases introduced with incorporating expert opinion, while the distribution represents the level of uncertainty about the treatment effect under investigation. The elicitation process is usually performed by the statistician of the trial asking more than one subject experts (i.e., physicians and their investigative teams) to report a few summaries of treatment effect, generally medians, modes, and percentiles of the probability distribution. Finally, a prior distribution is constructed by using a parametric method to match the available information. Sensitivity analyses with alternative assumptions are also important to ensure robustness. This is particularly useful when there is major disagreement between experts. Recently, the SHELF (Sheffield Elicitation Framework) [97] method is being used for formal prior elicitation by some sponsors [98] and may be a good choice in the rare disease setting as well. However, the time and cost needed to engage with subject-matter experts must be taken into consideration in the trial planning. Additionally, elicitation approaches may be best suited for internal decision-making and comparative effectiveness trials, but this has some limitations in the registration setting as the perceived level of validity of expert opinion that is not grounded in specific data.

Alternatively, another family of methods is to employ an informative prior based on available historical and external data as described in the case studies in “Case studies illustrating applicability and execution of Bayesian methods” section. External control information can come from different sources including randomized clinical trials, registries, electronic health record, etc. Use of an external control to augment a concurrent control group (or to replace concurrent control in settings where randomization is considered unethical) is ethically and operationally appealing. In this setting, a careful selection of the historical trials is necessary to ensure the exchangeability assumption for the control parameters is plausible. Pocock [99] proposed a set of criteria to assess the similarity between external control and trial control data that requires taking into account many different aspects (ICH E10 (2001)) [100]. For example, the Pocock criteria consider inclusion and exclusion criteria, the distribution of baseline characteristics, disease characteristics, treatment, relevant medical practice, endpoint definition, and follow-up between the external and current data. Thorlund et al. provided a checklist on similarity evaluation when using external controls in clinical trials [101]. The choice should not be biased by outcome of interest or actual analysis of candidate external control group data. This may happen for example if historical data/trials with superior results are preferentially omitted. As a result, it is important that the entire selection process of a dataset and patient-level data be pre-specified independent of outcome data. Nevertheless, one must acknowledge the possible conflict between external control and trial control due to unknown factors. For example, published studies do not always have detailed covariate information. Therefore, it is important to consider analysis approaches (e.g., mixture prior as illustrated in the PSP example in “Use of external control data in the designing phase III trial for Progressive Supranuclear Palsy (PSP)” section) to handle such conflicting scenarios.

When there is little information available on which to base the prior distribution, a natural choice is a non-informative or weakly informative prior. These are intended to represent a minimal amount of prior information. Although non-informative priors may yield similar results to the frequentist approach, the priors may be difficult to interpret because they attempt to assign equal weight to all values of interest. Nonetheless, there may be other advantages to using Bayesian methods to get the results. Other prior distribution alternatives include *skeptical prior distributions* and *enthusiastic prior distributions* that quantify the belief that a large treatment effect is unlikely or likely, respectively. As the true effect is unknown, sensitivity analyses to alternative prior assumptions are vital and should be an integral part of analysis to ensure the robustness of study results [102]. It is required to ensure that the prior does not have unintended influence on the inference and trial conclusion. Finally, it is preferable to construct a prior distribution on a scale that has a straightforward interpretation for the parameter of interest. This is helpful when communicating results with non-statisticians.

The FDA Complex Innovative Design interaction guidance emphasizes the need for sponsors to include a detailed description of the prior specification for transparency [39]. This includes the rationale for choosing source data and/or expert opinion, methods for prior derivation, and the impact on the analysis via simulation with hypothetical trial data.

#### Statistical computation

A common feature of Bayesian design and analyses is the use of simulations to estimate frequentist operating characteristics (e.g., type I error, power) or to evaluate design parameters (e.g., timing of interim analyses, futility probability). Simulations are computationally intensive; they are also a powerful tool to virtually explore thousands and thousands of potential trial scenarios to understand the overall behavior of the design. It is important to present the operating characteristics obtained from the simulations in the study protocol to maintain full transparency about design and analysis. Operating characteristics are also critical for studies intended to use Bayesian design; both the FDA complex innovative design interactions [39] and adaptive design [38] guidance suggest the need for trial simulations. Specialized computational algorithms are often required for Bayesian analysis to analyze trial data, assess prior probabilities at the design stage, perform simulations to assess the probabilities of various outcomes, and estimate sample size. Although Bayesian analyses are often computationally intensive, recent breakthroughs in computational algorithms and computing speed have made it possible to carry out calculations for very complex and realistic Bayesian models [103–107].

#### Communication

Implementing the proposed approach requires Bayesian expertise, which should include the ability to explain the approach in plain English, and technical skills related to concrete implementation [108, 109]. Discussing important features of the statistical approach with clinical colleagues is one of the key responsibilities of a statistician when designing and implementing clinical trials. This is even more important for trials using a Bayesian approach. Members of the clinical team may have no or very little experience with Bayesian designs or analyses, and it is therefore recommended that the main features of the approach be explained using visual illustrations and non-statistical language. Providing analysis results in both graphic and tabular form if often helpful. Examples include (1) figures of prior and posterior distribution showing reduction in uncertainty using simple language, and (2) a plot with the prior belief at the x-axis and corresponding posterior results on the y-axis to aid in understanding the impact of the choice of prior. Good preparation includes a mimicked trial analysis with different data scenarios to highlight various possible trial outcomes.

In a clinical trial, it is important that practitioners understand the risks and benefit of a new drug based on all available contextual information (clinical, pre-clinical, and non-clinical) at the design stage and how to interpret the result(s) at the end of the trial. However, many clinicians and scientists are not familiar with interpreting an effect size as a patient centric probabilistic statement induced by Bayesian statistics. This a major contrast compared to the frequentist approach where a treatment effect is communicated via a point estimate, confidence interval, and p-values. A survey conducted by the DIA Bayesian Scientific working group revealed that a lack of knowledge was the top barrier to implementing those methods more broadly [110]. The survey also revealed that in-person training was the top-ranked option and online training the second preference for helping non-statisticians become comfortable with using Bayesian statistics. This training needs to explain how an observed treatment effect can be converted into a probabilistic statement. Interactive software such as R-shiny applets or Fixed and Adaptive Clinical Trial Simulator (FACTS) [111] can provide illustrative examples highlighting the use of Bayesian statistics in clinical trials. In addition, it is important for the training program to include a wide range of case studies from rare diseases to demonstrate the application and use of Bayesian methods to non-statisticians.

#### Regulatory perspectives

The FDA and European Medicines Agency (EMA) explicitly recognize the utility of Bayesian methods in rare disease [37–39, 112, 113]. While the use of Bayesian methods is appealing for practitioners, careful consideration should be given to near-term uses in appropriate regulatory and clinical contexts. For example, external control data may be used to augment randomized control arms as part of a hybrid approach (i.e. as opposed to a single experimental arm trial with only historical/external controls or a randomized clinical trial considering data only from concurrent controls) that could reduce the number of patients that are randomized to the control arm within a study. Given a solid rationale for an external control, and a careful assessment of whether an external control would be scientifically feasible, the actual implementation of the external control requires care and planning [114].

Several procedural best practices are advised to ensure robust and credible implementation of novel Bayesian approaches. Pre-specification of protocols and statistical analysis plans provide confidence that the plan could be independently performed or duplicated, and at a minimum should include:

- A detailed protocol with clear objectives and description of the study population, as well as details regarding data sources and critical features of the study design and analysis plan.
- The methodological approach should be specified in the statistical analysis plan or other companion document.
- The final statistical analysis and any sensitivity analyses should be clearly pre-specified and consistent with good statistical practice.

Early discussions with regulators and review of key planning documents can often result in valuable feedback for sponsors using Bayesian approaches. Sponsors should consider soliciting regulatory feedback by means of protocol submissions or formal product meetings. For US regulatory feedback, sponsors may also explore opportunities for participation in the FDA’s Complex Innovative Trial Designs (CID) pilot program [115], which exists to further the use of new trial designs. A recent publication details several Bayesian trial designs reviewed under the CID pilot program, covering a range of possibilities discussed in this article including platform designs, complex adaptive designs, and external controls [116].

On January 18th, 2022, FDA published three case studies from the CID pilot program [115], which include aspects of the study design, summaries of the trial simulations performed, and discussions of the regulatory implications of the design and analyses. One of the case studies is a randomized, double-blind, phase 2 trial in patients with systemic lupus erythematosus (SLE), a rare disease with a high unmet need. Patients were assigned to one of four treatment groups: three different doses of the product or a placebo. The study pooled data from different dose levels in comparing the treatment to the placebo and used Bayesian methods to estimate response rates across treatment groups. The other two examples include a master protocol in chronic pain and a randomized phase III study in diffuse B-cell lymphoma (DLBCL), both of which borrowed information from external control data and used Bayesian methods for the design and analysis. Furthermore, the CID pilot program update mentions another randomized, double-blind, placebo-controlled study in ambulatory patients with Duchenne Muscular Dystrophy using Bayesian repeated measures and augmenting trial data with historical placebo data for analysis [116]. The case studies note several key points that arose in interactions between sponsors and FDA on these Bayesian designs, including addressing exchangeability, ensuring the parameter space explored in simulations was adequately broad (including for nuisance parameters), and the appropriate use of propensity scores in models that borrow external control information.

## Conclusion

The rarity of patients is the overarching challenge for conducting clinical studies for rare diseases. Thus, it is sometimes infeasible to use traditional clinical trial design and analytic methods to obtain the substantial evidence of effectiveness regulatory agencies need to approve products for rare diseases. The use of innovative trial designs such as master protocols and complex adaptive designs in conjunction with a Bayesian approach may help to reduce sample size, choose the correct treatment and population, and accurately and reliably assess the treatment effect in the rare disease setting. Additional applications of the design and methods included in this manuscript include the extrapolation of adult data to a pediatric population and the assessment of subgroup-specific treatment effects in rare diseases.

Beyond Bayesian design of individual trials, Bayesian methods for meta-analysis are appealing in rare diseases as they can increase the precision of a treatment effect estimate by borrowing information from all available sources of clinical evidence including early phase trials. There are some specific challenges for meta‐analyses in small populations and rare diseases. Trials with small sample size pose more between-trial variability due to the difference in controls used, disease characteristics, and treatment allocation (randomized vs. non-randomized). Use of data sources other than from randomized controlled trials can generate biased treatment effect estimates, which poses further challenges to the interpretation of the meta-analyses. One solution is the use of generalized evidence synthesis techniques [97, 117]. This is an extension of the standard random-effects meta-analysis model incorporating a third level in the hierarchy to account for heterogeneity between study design types explicitly, as well as heterogeneity between individual studies with the same design.

The use of Bayesian methods and nontraditional study designs to inform regulatory approval of new drugs or expanded indications of marketed drugs is an exciting and active area of research. Ultimately, we agree with Ruberg [118] that Bayesian and frequentist approaches can exist harmoniously in clinical trial design and inference such that frequentist operating characteristics can be shown to be met in a Bayesian design, but a Bayesian analysis and interpretation may be most appropriate and advantageous, especially in rare disease settings. The fundamental goal for all the innovative trial designs and Bayesian methods presented is to effectively use all available data to make robust, accurate and timely decisions about the risk–benefit profile of a drug. These, as well as improvements in quality of data, will likely increase in the hierarchy of evidence-based investigation and help advance the development of innovative medicines for patients with rare disease.

## Supplementary Information

**Additional file 1.** Use of external control data in the designing phase III trial for Progressive Supranuclear Palsy (PSP).

## Data Availability

Simulation code available in appendix.

## Abbreviations

FDA: Food and Drug Administration
PDUFA: Prescription Drug User Fee Act
PSP: Progressive Supranuclear Palsy
PSPRS: Progressive Supranuclear Palsy Rating Scale
MAP: Meta-analytic predictive
SMART: Sequential, multiple assignment, randomized trial
snSMART: Small sample (n) sequential, multiple assignment, randomized trial
InPACT: International Penile Advanced Cancer Trial
ARAMIS: A RAndomized Multicenter study for isolated skin vasculitis
INHIBIT: Platform clinical trial composed of 2 phase 3 randomized trials: the Inhibitor Prevention Trial and the Inhibitor Eradication Trial
DIAN-TU: Dominantly Inherited Alzheimer Network Trials Unit
SHELF: Sheffield elicitation framework
EMA: European Medicines Agency
CID: Complex Innovative Design
