# Supplementary material for: Application of Bayesian methods to accelerate rare disease drug development: scopes and hurdles
Source: Orphanet J Rare Dis. 2022 May 7;17:186. doi: 10.1186/s13023-022-02342-5 (PMC9077995; doi:10.1186/s13023-022-02342-5)
Supplement: Supplementary file 1 — Additional file 1. Use of external control data in the designing phase III trial for Progressive Supranuclear Palsy (PSP). [file 13023_2022_2342_MOESM1_ESM.pdf]

Appendix: R Code for Case Study 3.1

This is an R Markdown document.

```
#load required library

library(RBesT)
```

```
## Warning: package 'RBesT' was built under R version 4.1.2
```

```
## This is RBesT version 1.6.3
```

```
library(MASS)
library(ggplot2)
library(gridExtra)
library(grid)
library(ggpubr)
```

```
## Warning: package 'ggpubr' was built under R version 4.1.2
```

```
#Historical Placebo data

y.mean <- c(10.9, 11.4, 10.5)
y.sd <- c(11.0, 6.3, 7.6)
n <- c(153, 31, 59)
study=c("Boxer","Tolosa", "Höglinger")

hist.data.PSP <- data.frame(y.m=y.mean, y.se = y.sd/sqrt(n), n=n, study=study)


sd.ctl <- 8


set.seed(1234)

PSP_map_mcmc <- gMAP(cbind(y.m, y.se) ~ 1 | study,
                     weights=n,data=hist.data.PSP,
                     family=gaussian,
                     beta.prior =10,
    tau.dist="HalfNormal",tau.prior=cbind(0,sd.ctl/6))
```

```
## Assuming default prior location   for beta: 0
```

```
print(PSP_map_mcmc)
```

```
## Generalized Meta Analytic Predictive Prior Analysis
##
## Call:  gMAP(formula = cbind(y.m, y.se) ~ 1 | study, family = gaussian,
##     data = hist.data.PSP, weights = n, tau.dist = "HalfNormal",
##     tau.prior = cbind(0, sd.ctl/6), beta.prior = 10)
##
## Exchangeability tau strata: 1
```

```
## Prediction tau stratum    : 1
## Maximal Rhat              : 1
## Estimated reference scale : 8.9
##
## Between-trial heterogeneity of tau prediction stratum
##   mean     sd   2.5%    50%  97.5%
## 0.6840 0.5680 0.0271 0.5420 2.1300
##
## MAP Prior MCMC sample
##  mean    sd  2.5%   50% 97.5%
## 10.80  1.18  8.46 10.80 13.20
```

```
PSP_map_mcmc.data <- forest_plot(PSP_map_mcmc)$data

#Using method of moment approximation

PSP_map_mcmc.mix <- mixfit(PSP_map_mcmc, Nc=1)


print(PSP_map_mcmc.mix)
```

```
## EM for Normal Mixture Model
## Log-Likelihood = -6329.066
##
## Univariate normal mixture
## Reference scale: 8.901176
## Mixture Components:
##   comp1
## w  1.000000
## m 10.848092
## s  1.183057
```

```
plot(PSP_map_mcmc.mix)$mix
```

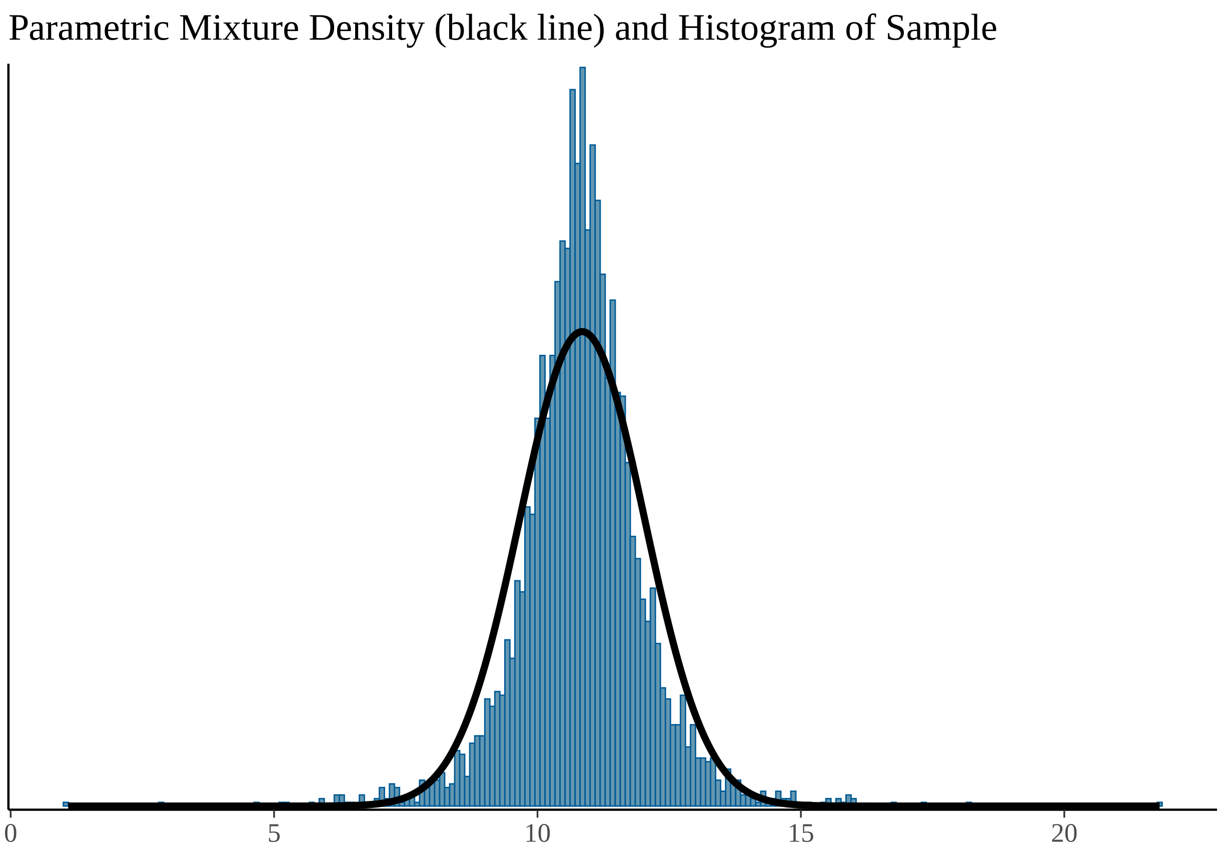


```
#Alternatively, one can do mixture approximation of the predictive distribution with lowest AIC

PSP_map_mcmc.bmix <- automixfit(PSP_map_mcmc)


print(PSP_map_mcmc.bmix)
```

```
## EM for Normal Mixture Model
## Log-Likelihood = -6016.492
##
## Univariate normal mixture
## Reference scale: 8.901176
## Mixture Components:
##   comp1       comp2       comp3
## w  0.60908219  0.34258384  0.04833397
## m 10.83116507 10.89045907 10.76110759
## s  0.69015905  1.41054267  2.97134789
```

```
plot(PSP_map_mcmc.bmix)$mix
```

Parametric Mixture Density (black line) and Histogram of Sample

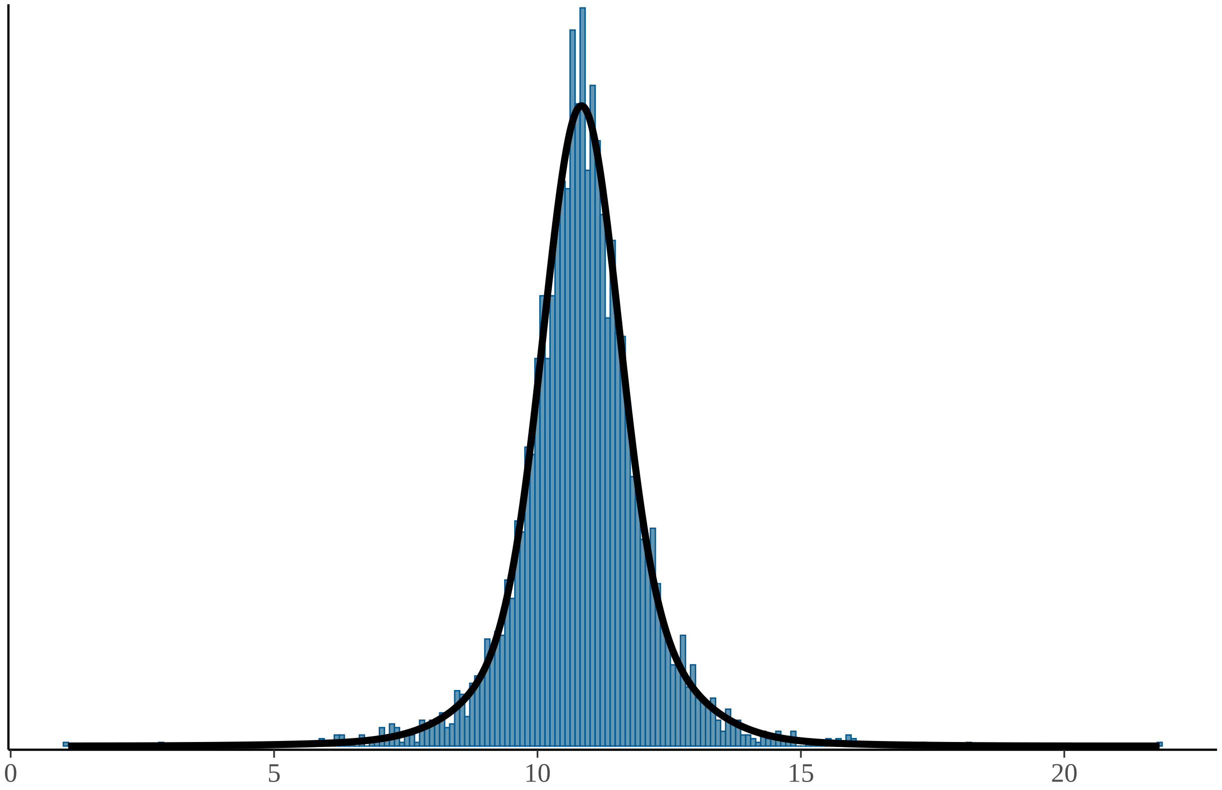


```r
ggplot(PSP_map_mcmc.data, aes(x=study, y=median, ymin=low, ymax=up, linetype=model, shape=model)) +
  geom_pointrange(size=0.7, position=position_dodge(width=0.5)) +
  geom_hline(yintercept=qmix(PSP_map_mcmc.mix, 0.5), linetype=3, alpha=0.5) +
  coord_flip() + theme_bw(base_size=12) + theme(legend.position="None") +
  labs(x="", y="Mean PSPRS Change at week 52", title="")
```

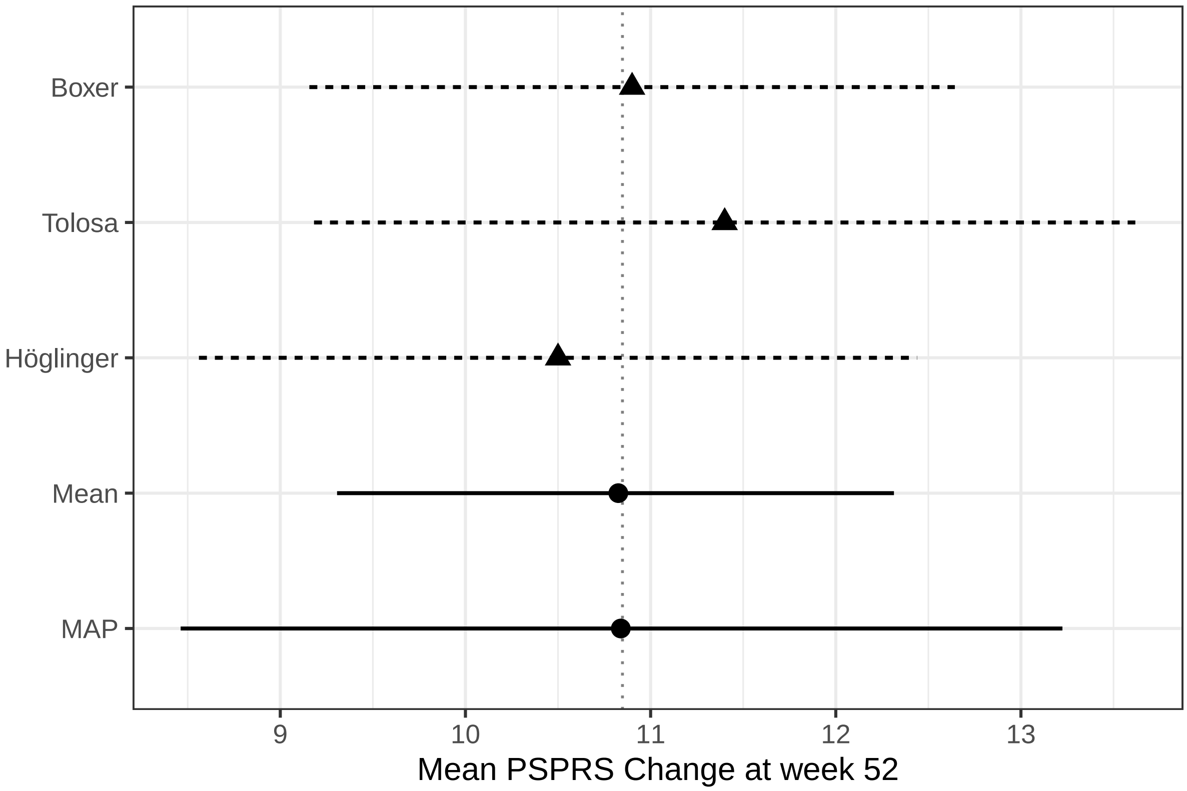


```
#Robustify MAP prior

Rob_PSP_map_mcmc.mix <- robustify(PSP_map_mcmc.mix, weight=0.5, mean=15, sigma=10)
```

```
#Operating Characteristics

succc <- decision2S(pc=0.975, qc=0, lower.tail=FALSE)
print(succ)
```

```
## 2 sample decision function
## Conditions for acceptance:
## P(x1 - x2 > 0) > 0.975
## Link: identity
```

```
#Design specification

#Weakly informative prior for treatment group
weak_prior <- mixnorm(c(1,15,1), sigma=10, param = 'mn')


n.act <- 85
n.pbo <- 43


# the range for true values for the placebo group
theta_truth <- seq(8,20, by=0.1)
```

```r
## Two designs: One with MAP prior and other with Robust MAP
#Design 1: weak prior for treatment and MAP prior for control
#Design 2: weak prior for treatment and robust MAP prior for control

design1  <- oc2S(weak_prior, PSP_map_mcmc.mix , n.act, n.pbo, succ, sigma1=sd.ctl, sigma2=sd.ctl)
design2 <- oc2S(weak_prior, Rob_PSP_map_mcmc.mix , n.act, n.pbo, succ, sigma1=sd.ctl, sigma2=sd.ctl)

#Type-I error

typeI.design1 <- design1(theta_truth, theta_truth)
typeI.design2 <- design2(theta_truth, theta_truth)


oc.TI <- rbind(data.frame(theta_truth=theta_truth, typeI=typeI.design1,
                          Design="MAP prior"),
               data.frame(theta_truth=theta_truth, typeI=typeI.design2,
                          Design="Robust MAP prior")
)



#Power

delta <- 4
m <- seq(8,20, by=0.1)

hist.m <- summary(PSP_map_mcmc)$theta.pred[,"mean"]

theta.truth.act <- m + delta   # active
theta.truth.pbo <- m + 0*delta # pbo


power.design1 <- design1(theta.truth.act, theta.truth.pbo)
power.design2 <- design2(theta.truth.act, theta.truth.pbo)


oc.P <- rbind(data.frame(theta_truth_a=theta.truth.act, theta_truth_p=theta.truth.pbo, delta= delta,
                         power=power.design1, Design="MAP prior"),
              data.frame(theta_truth_a=theta.truth.act, theta_truth_p=theta.truth.pbo, delta= delta,
                         power=power.design2, Design="Robust MAP prior")
)

p1 <- qplot(theta_truth, typeI, data=oc.TI, linetype = Design, geom="line",
           main=expression(paste("Type I Error (", delta, "=0)"))) +
  geom_line(aes(linetype=Design))+
  xlab('True Mean Change for Placebo') +
  ylab('Type I error') +
  coord_cartesian(ylim=c(0,1)) + geom_hline(yintercept=0.025, linetype="dashed", color="gray32") +
  theme(plot.title = element_text(hjust = 0.5), panel.background = element_blank(),
        panel.grid.major = element_blank(),
        panel.grid.minor = element_blank(),
        axis.line = element_line(colour = "black"),
        panel.border = element_rect(colour = "black",
```

```
                                      fill=NA, size=1))

p2 <- qplot(theta_truth_p, power, data=oc.P, linetype=Design, geom="line",
            main=expression(paste("Power (", delta, "=4)"))) +
  theme(plot.title = element_text(hjust = 0.5),
        panel.background = element_blank(),
        panel.grid.major = element_blank(),
        panel.grid.minor = element_blank(),
        axis.line = element_line(colour = "black"),
        panel.border = element_rect(colour = "black", fill=NA, size=1))+
  geom_line(aes(linetype=Design))+
   xlab('True Mean Change for Placebo') +
  ylab('Power') +
  geom_vline(xintercept = hist.m, lty=3)+
  geom_hline(yintercept=0.77, linetype="dashed", color="gray32") +
  coord_cartesian(ylim=c(0,1))

p <- ggarrange(p1, p2, ncol=2, nrow=1, common.legend = TRUE, legend="bottom")

p
```

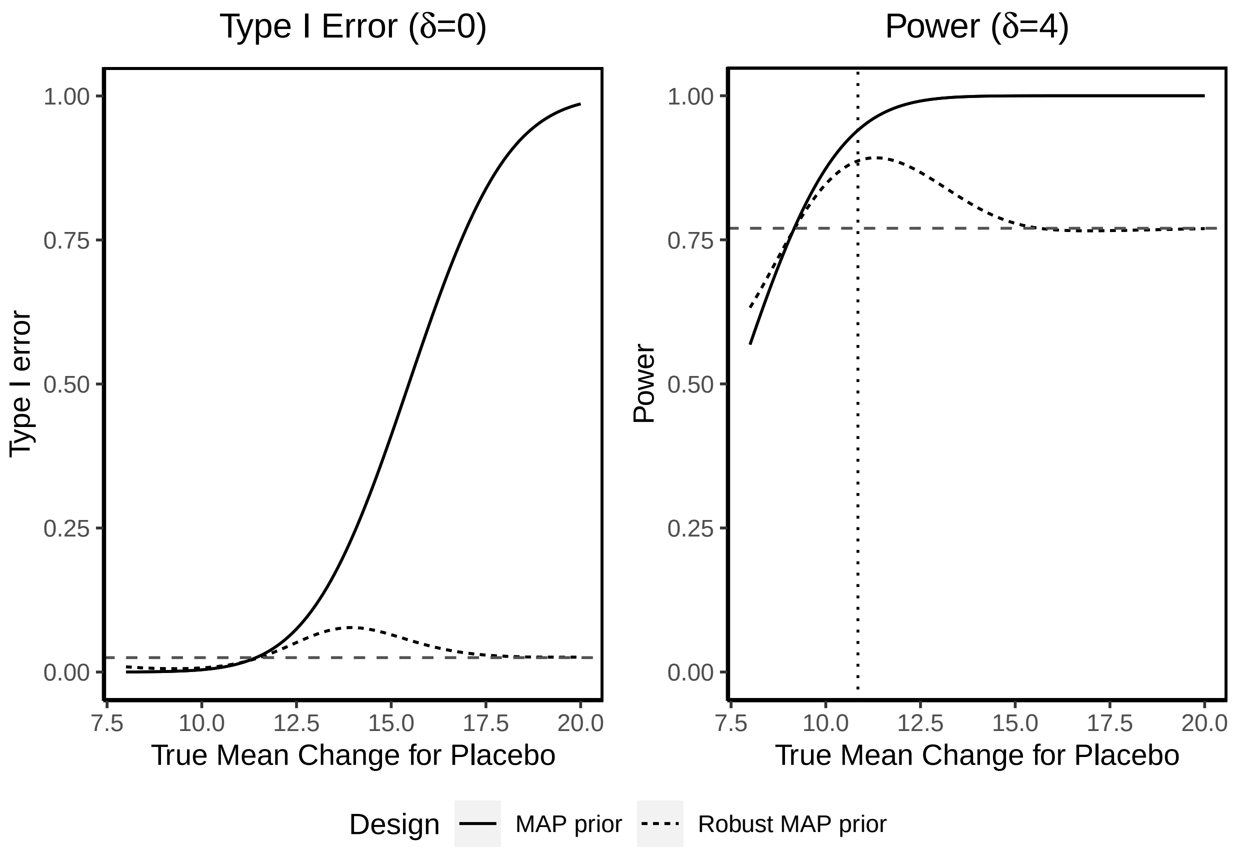


```
sessionInfo()
```

```
## R version 4.1.0 (2021-05-18)
## Platform: x86_64-w64-mingw32/x64 (64-bit)
## Running under: Windows 10 x64 (build 17134)
```

```
##
## Matrix products: default
##
## locale:
## [1] LC_COLLATE=English_United States.1252
## [2] LC_CTYPE=English_United States.1252
## [3] LC_MONETARY=English_United States.1252
## [4] LC_NUMERIC=C
## [5] LC_TIME=English_United States.1252
##
## attached base packages:
## [1] grid      stats     graphics  grDevices utils     datasets  methods
## [8] base
##
## other attached packages:
## [1] ggpubr_0.4.0  gridExtra_2.3 ggplot2_3.3.5 MASS_7.3-54   RBesT_1.6-3
##
## loaded via a namespace (and not attached):
##  [1] tidyr_1.1.3          jsonlite_1.7.2       carData_3.0-4
##  [4] RcppParallel_5.1.4   StanHeaders_2.21.0-7 Formula_1.2-4
##  [7] assertthat_0.2.1     highr_0.9            stats4_4.1.0
## [10] yaml_2.2.1           pillar_1.6.1         backports_1.2.1
## [13] glue_1.4.2           digest_0.6.27        ggsignif_0.6.3
## [16] checkmate_2.0.0      colorspace_2.0-2     cowplot_1.1.1
## [19] htmltools_0.5.1.1    plyr_1.8.6           pkgconfig_2.0.3
## [22] rstan_2.21.2         broom_0.7.9          purrr_0.3.4
## [25] mvtnorm_1.1-2        scales_1.1.1         processx_3.5.2
## [28] tibble_3.1.2         bayesplot_1.8.1      generics_0.1.0
## [31] farver_2.1.0         car_3.0-12           ellipsis_0.3.2
## [34] withr_2.4.2          cli_3.0.1            magrittr_2.0.1
## [37] crayon_1.4.1         evaluate_0.14        ps_1.6.0
## [40] fansi_0.5.0          rstatix_0.7.0        pkgbuild_1.2.1
## [43] tools_4.1.0          loo_2.4.1            prettyunits_1.1.1
## [46] lifecycle_1.0.0      matrixStats_0.61.0   stringr_1.4.0
## [49] V8_3.6.0             munsell_0.5.0        callr_3.7.0
## [52] compiler_4.1.0       rlang_0.4.11         ggridges_0.5.3
## [55] rstudioapi_0.13      labeling_0.4.2       rmarkdown_2.10
## [58] gtable_0.3.0         codetools_0.2-18     inline_0.3.19
## [61] abind_1.4-5          DBI_1.1.1            curl_4.3.2
## [64] reshape2_1.4.4       R6_2.5.0             rstantools_2.1.1
## [67] knitr_1.36           dplyr_1.0.7          utf8_1.2.1
## [70] stringi_1.6.2        parallel_4.1.0       Rcpp_1.0.7
## [73] vctrs_0.3.8          tidyselect_1.1.1     xfun_0.24
```
